# Correction: Design and synthesis of eugenol/isoeugenol glycoconjugates and other analogues as antifungal agents against *Aspergillus fumigatus*

**DOI:** 10.1039/d4md90005g

**Published:** 2024-02-12

**Authors:** Lakshmi Goswami, Lovely Gupta, Sayantan Paul, Maansi Vermani, Pooja Vijayaraghavan, Asish K. Bhattacharya

**Affiliations:** a Division of Organic Chemistry, CSIR-National Chemical Laboratory (CSIR-NCL) Dr. Homi Bhabha Road Pune 411 008 India ak.bhattacharya@ncl.res.in; b Academy of Scientific and Innovative Research (AcSIR) Ghaziabad 201 002 India; c Antimycotic and Drug Susceptibility Laboratory, J3 Block, Amity Institute of Biotechnology, Amity University Uttar Pradesh Sector-125 Noida India vrpooja@amity.edu

## Abstract

Correction for ‘Design and synthesis of eugenol/isoeugenol glycoconjugates and other analogues as antifungal agents against *Aspergillus fumigatus*’ by Lakshmi Goswami *et al.*, *RSC Med. Chem.*, 2022, **13**, 955–962, https://doi.org/10.1039/D2MD00138A.

The authors regret the following errors in their article on page 956 (right column, 3 lines above “Results and discussion” in the sentence starting “In addition, the incorporation…”, and 3 lines below “Results and discussion” in the sentence starting “We report herein the design…”). Both references to “the 1,2,4-triazole ring” should read instead: “the 1,2,3-triazole ring”.

The Royal Society of Chemistry apologises for these errors and any consequent inconvenience to authors and readers.

## Supplementary Material

